# *ADH1C* Ile350Val Polymorphism and Cancer Risk: Evidence from 35 Case–Control Studies

**DOI:** 10.1371/journal.pone.0037227

**Published:** 2012-05-18

**Authors:** Yao Xue, Meilin Wang, Dongyan Zhong, Na Tong, Haiyan Chu, Xiaojing Sheng, Zhengdong Zhang

**Affiliations:** 1 State Key Laboratory of Reproductive Medicine, Institute of Toxicology, Nanjing Medical University, Nanjing, China; 2 Department of Molecular & Genetic Toxicology, the Key Laboratory of Modern Toxicology of Ministry of Education, School of Public Health, Nanjing Medical University, Nanjing, China; 3 Department of Occupational Medicine and Environmental Health, Jiangsu Key Laboratory of Cancer Biomarkers, Prevention and Treatment, Cancer Center, Nanjing Medical University, Nanjing, China; Institut Jacques Monod, France

## Abstract

**Background:**

Alcohol dehydrogenase 1C (ADH1C) is the key enzyme catalyze oxidation of alcohol to acetaldehyde, which plays vital roles in the etiology of various cancer. To date, studies investigated the association between a functional polymorphism in *ADH1C*, Ile350Val (rs698), and risk of cancer have shown inclusive results.

**Methods:**

A meta-analysis based on 35 case-control studies was performed to address this issue. Odds ratios (OR) with 95% confidence intervals (CIs) were used to assess the association. The statistical heterogeneity across studies was examined with χ2-based Q-test.

**Results:**

Overall, no significant associations between ADH1C Ile350Val polymorphism and cancer risk were observed in any genetic models (P>0.05). In the stratified analyses, there was a significantly increased cancer risk among African (Val/Val vs. Ile/Ile OR  = 2.19, 95% CI  = 1.29−3.73, *P*_heterogeneity_  = 0.989; Ile/Val + Val/Val vs. Ile/Ile: OR  = 1.79, 95%CI  = 1.18−2.71, *P*_heterogeneity_  = 0.761; Val/Val vs. Ile/Val + Ile/Ile: OR  = 1.92, 95% CI  = 1.16−3.17, *P*_heterogeneity_  = 0.981) and Asian (Ile/Val vs. Ile/Ile: OR  = 1.58, 95% CI  = 1.32−1.90, *P*_heterogeneity_  = 0.375; Val/Val vs. Ile/Ile: OR  = 3.84, 95% CI  = 1.74−8.49, *P*_heterogeneity_  = 0.160; Ile/Val + Val/Val vs. Ile/Ile: OR  = 1.65, 95% CI  = 1.38−1.96, *P*_heterogeneity_  = 0.330; Val/Val vs. Ile/Val + Ile/Ile: OR  = 3.54, 95% CI  = 1.62−7.75, *P*_heterogeneity_  = 0.154) studies.

**Conclusions:**

The results indicate that *ADH1C* Ile350Val polymorphism may contribute to cancer risk among Africans and Asians. Additional comprehensive system analyses are required to validate this association combined with other related polymorphisms.

## Introduction

There has been convincing evidence that alcohol ingestion is carcinogenic to humans and causally related to liver, colorectal, breast and upper aerodigestive tract (UADT) cancers [1]. Although multiple mechanisms are involved in alcohol-mediated carcinogenesis, it has been shown that acetaldehyde (AA), the oxidative product of ethanol (commonly called alcohol), rather than alcohol itself is the principal carcinogenic material in alcohol metabolism [2]. AA interferes at many sites with DNA synthesis and repair and consequently has direct mutagenic and carcinogenic effects [3]. The key enzyme responsible for oxidation of ethanol to AA is alcohol dehydrogenase (ADH) [4]. Human ADH family is a well-defined system of enzymes which play important role in detoxification of alcohols and are categorized into several classes based on differences in substrate specificity, sensitivity to inhibitors, localization, electrophoretic migration and immunological properties [5]. In addition to the first-pass ethanol metabolism, ADH has shown various functions including activity towards hydroxysteroids, detoxification of endogenous and exogenous formaldehyde, retinoid transformation, etc. [6], [7], [8]. The differences of the activities of total ADH and ADH isoenzymes between cancer and healthy tissue have been demonstrated [4]. As production rate of AA is mainly modulated by ADH, it is rational that ADH activity variation may have effects on the level of AA in vivo and be one of the factors intensifying carcinogenesis.

There are seven genes that encode the seven known isozymes of human ADH. According to structural characteristics, the seven isozymes are categorized into five different classes, among which Class I isozymes account for most of the alcohol metabolism [9]. The three class I genes, *ADH1A*, *ADH1B*, and *ADH1C* (formerly known as *ADH1*, *ADH2* and *ADH3*) are very closely related; they encode alpha (α), beta (β) and gamma (γ) subunits, respectively [10]. Functional variants (i.e., single nucleotide polymorphisms, SNPs) arousing wide concern exist in two of three genes encoding ADH enzymes (i.e., the *ADH1B* and *ADH1C* genes) [11]. The polymorphic sites for *ADH1B* are Arg48His in exon 3 (rs1229984) and Arg370Cys in exon 9 (rs2066702) and for *ADH1C* are Arg272Gln (rs1693482) and Ile350Val (rs698) [10]. The ADH1B*1 allele is a name for the reference allele encoding β1 subunit which has arginine (Arg) at positions 48 and 370. ADH1B*2 (β2) refers to a variant allele defined by histidine (His) at position 48 while ADH1B*3 encoding β3 subunit that has cysteine (Cys) at position 370 [10]. For polymorphisms in *ADH1C*, 272Arg and 350Ile carriers have the ADH1C*1 allele, whereas 272 Gln and 350 Val carriers have the ADH1C*2 allele [12]. It is worth noting that significant linkage disequilibrium has been detected between the *ADH1B* and *ADH1C* polymorphisms as well as the two variants in *ADH1C* [13], [14]. These functional variants result in the production of enzymes with different kinetic properties [10], [15] and subsequently the generation of different quantities of AA. For example, individuals with ADH1C*1 allele have an ethanol oxidizing capacity 2.5-times higher when compared to ADH1C*2 allele [12]. Thus, not only the amount of alcohol is determinant for organ injury, but also the genetic factors may modulate and determine carcinogenesis.

An increasing number of studies have investigated the association between *ADH* polymorphisms and cancer risk in human. Among them, studies of *ADH1C* Ile350Val variant accounted for more than others. Most of the *ADH1C* studies focused on head and neck cancer (HNC) development, and to a less extent on the cancers of breast, colorectum, etc. Although genotype frequency of Ile350Val polymorphism varies among different populations [16], evidences supporting the association between this genetic variant and risk of cancer have arisen from studies of different ethnic background [17], [18], [19]. Recently, Chang *et al.* conducted a meta-analysis to assess the association between *ADH1B* and *ADH1C* polymorphisms and risk of HNC [20], and they found a reduced risk for HNC associated with ADH1B*2 and ADH1C*1 alleles. However, as the studies on *ADH1C* polymorphism and different cancer risk have shown contradictory and inconclusive results, a pooled analysis of all studies on *ADH1C* and cancer risk is needed.

Here, we performed a meta-analysis on 35 eligible case-control studies to estimate the overall cancer risk and *ADH1C* polymorphisms. Because polymorphisms of Arg272Gln and Ile350Val were in strong linkage disequilibrium and both of them can be used to distinguish ADH1C*1 and ADH1C*2 alleles, we focused on the most commonly studied polymorphism Ile350Val.

## Materials and Methods

### Identification and Eligibility of Relevant Studies

PubMed and EMBASE were searched for all relevant reports (the last search update was July 18, 2011), using the search terms “ADH1C” or “ADH3”, “polymorphism” and “cancer”. The search was limited to English language papers. In addition, studies were identified by a manual search of the references of original studies. Of the articles with the overlapping data, we only selected the publication with the most extensive information. For inclusion in the meta-analysis, the identified articles had to meet the following criteria: (a) there were information on the evaluation of the *ADH1C* Ile350Val polymorphism and cancer risk, (b) used a case–control design, and (c) contained complete information about all genotype frequency. The exclusion criteria were as follows: (a) not for cancer research, (b) review articles, (c) reports without usable data and (d) duplicate publications.

### Data Extraction

Two authors (Y Xue and M Wang) extracted data from all eligible publications independently and reached a consensus on all the items. For each study, the following characteristics were considered: the first author’s last name, year of publication, country of origin, ethnicity, cancer type, source of control groups (population- or hospital-based controls) and numbers of genotyped cases and controls. Different ethnic descents were categorized as African, Asian, European, or Mixed (composed of different ethnic groups). Cancers of oral cavity, oropharynx, hypopharynx, larynx, esophagus and stomach were defined as upper aerodigestive tract (UADT) cancers [21], [22]. For studies including subjects of different ethnic groups or cancer types, data were extracted separately for each ethnic group or cancer type whenever possible.

### Statistical Analysis

The strength of the association between the *ADH1C* Ile350Val polymorphisms and cancer risk was measured by odds ratios (ORs) with their 95% confidence intervals (CIs). The statistical significance of the summary OR was determined with the Z-test. We first explored the risks of the Ile/Val and Val/Val genotypes on cancer, compared with the wild-type Ile/Ile homozygote, and then evaluated the risks of Ile/Val + Val/Val versus Ile/Ile and Val/Val versus Ile/Val + Ile/Ile on cancer, assuming dominant and recessive effects of the variant Val allele, respectively. Stratified analyses were also performed by cancer types (if one cancer type contained less than three individual studies, it was classified as other cancers group), ethnicity, source of controls and sample size (subjects >500 in both case and control groups or not).

In consideration of the possibility of heterogeneity across the studies, a statistical test for heterogeneity was performed by a χ^2^-based Q-test. A *P*-value greater than 0.10 for the Q-test indicated lack of heterogeneity among the studies, and then the fixed-effects model (the Mantel–Haenszel method) was used to calculate the summary OR estimate of each study. Otherwise, the random-effects model (DerSimonian and Laird method) was used. Sensitivity analyses were performed to assess the stability of the results, namely, a single study in the meta-analysis was deleted each time to reflect the influence of the individual data set to the pooled OR. The presence of publication bias indicates that non-significant or negative findings remain unpublished. We used Funnel plots and Egger’s linear regression test to provide diagnosis of the potential publication bias. All statistical analyses were performed with the Stata software (version 8.2; StataCorp LP, College Station, TX, USA), using two-sided *P*-values.

## Results

### Characteristics of Studies

**Figure 1:**
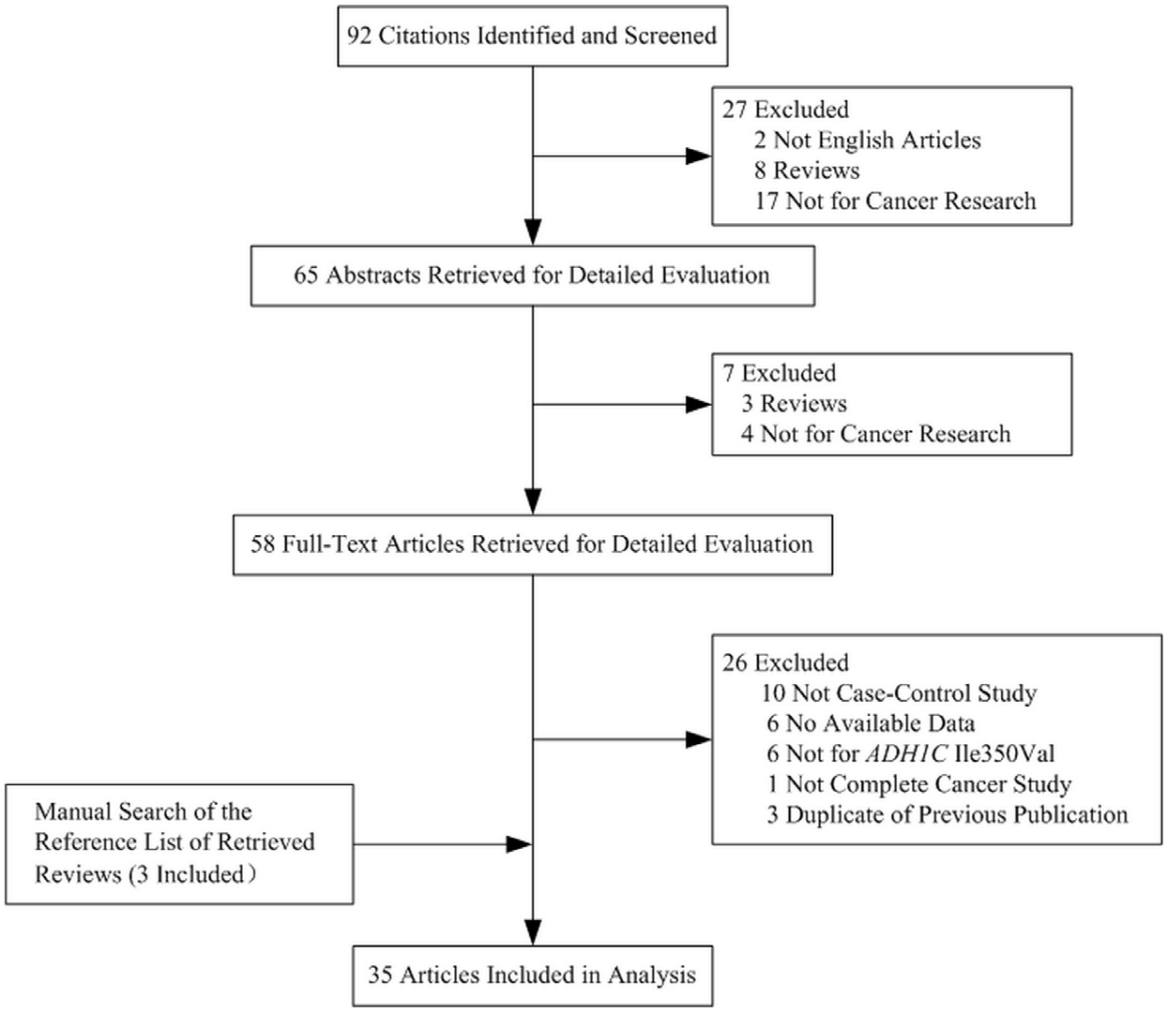
Studies identified with criteria for inclusion and exclusion.

There were 35 studies retrieved on the basis of the search criteria for cancer susceptibility associated with *ADH1C* Ile350Val polymorphisms (Fig. 1). Totally, 19,154 cases and 26,519 controls were included in the meta-analysis. Study characteristics are summarized in Table 1. Among the 35 case–control studies, there were 5 studies of Asians, 19 studies of Europeans and 8 studies of mixed descendents. Besides, 3 studies included more than one ethnic group [14], [23], [24]. Thus, in total, 2 African groups, 5 Asian groups, 21 European groups and 10 groups of mixed descendents were recruited in our analyses. Controls were mainly matched on sex and age, of which 14 were population based [21], [24], [25], [26], [27], [28], [29], [30], [31], [32], [33], [34], [35], [36], 16 were hospital based [17], [18], [19], [23], [37], [38], [39], [40], [41], [42], [43], [44], [45], [46], [47], [48] and 5 studies was conducted on both population-based and hospital-based control group [14], [22], [49], [50], [51]. Furthermore, 7 studies were conducted with subjects >500 in both case and control groups [14], [26], [28], [29], [34], [38], [51]. There were 4 studies of breast cancer, 25 of UADT cancer, 3 of colorectal and 3 of other cancers. Among the 25 UADT cancer studies, Homann *et al*. investigated the *ADH1C* polymorphism and cancer risk in both UADT cancer and hepatocellular cancer groups [50]. Thus, number of studies of “other cancers” was 4. Cancers were confirmed histologically or pathologically in most studies. The distribution of genotypes in the controls of all studies was consistent with Hardy–Weinberg equilibrium (HWE) except for 10 studies, 9 of which did not mention the HWE test [24], [31], [33], [36], [37], [41], [44], [48], [49] and in one study allele distributions were not in HWE for a part of controls [43].

**Table 1:** Characteristics of studies included in the meta-analysis.

First author	Year	Country	Ethnicity	Cancer types	Source of controls	Sample size	
						Case	Control
Coutelle	1997	France	European	UADT	HB	39	37
Harty	1997	Puerto Rico	Mixed	UADT	PB	146	146
Freudenheim	1999	USA	European	Breast	PB	315	356
Bouchardy	2000	France	European	UADT	HB	244	167
Chao	2000	China	Asian	UADT	HB	88	434
Olshan	2001	USA	European and African	UADT	HB	173	194
Sturgis	2001	USA	European	UADT	HB	229	575
Schwartz	2001	USA	European	UADT	PB	333	541
Dijk	2001	Netherlands	European	Bladder	HB	115	131
Zavras	2002	Greece	European	UADT	HB	93	99
Yokoyama	2002	Japan	Asian	UADT	PB	234	634
Freudenheim	2003	USA	Mixed	Lung	PB	113	212
Nishimoto	2004	Brazil	Mixed	UADT	Combined	141	134
Coutelle	2004	German	European	Breast	HB	117	111
Peters	2005	USA	European	UADT	PB	521	599
Wang	2005	USA	European	UADT	HB	348	330
Homann	2006.	German	European	UADT and Hepatocellular	Combined	293	729
Logt	2006	Netherlands	European	Colorectal	PB	320	385
Terry	2006	USA	Mixed	Breast	PB	1047	1101
Terry	2007	USA	Mixed	UADT	PB	197	160
Zhang	2007	Poland	European	UADT	PB	297	425
Yin	2007	Japan	Asian	Colorectal	PB	685	777
Visvanathan	2007	USA	European	Breast	PB	303	312
Asakage	2007	Japan	Asian	UADT	HB	96	642
Curtin	2007	USA	Mixed	Colorectal	PB	915	1969
Solomon	2008	India	Mixed	UADT	HB	126	100
Hashibe	2008	Multi-Countries	European and Mixed	UADT	Combined	3393	4851
Li	2008	South Africa	African and Mixed	UADT	PB	237	268
Oze	2009	Japan	Asian	UADT	HB	585	1170
Garcia	2010	Brazil	Mixed	UADT	HB	207	244
Duchonova	2010	Czech	European	Pancreatic	HB	235	264
Kortunay	2010	Turkey	European	UADT	HB	50	100
Soucek	2010	Slav	European	UADT	HB	121	121
Brocic	2011	Serbia	European	UADT	Combined	123	177
Mckay	2011	Multi-Countries	European	UADT	Combined	6675	8024

UADT, upper aerodigestive tract; HB, hospital based; PB, population based; Combined, studies conducted on both population-based and hospital-based control group.

### Quantitative Synthesis

**Figure 2:**
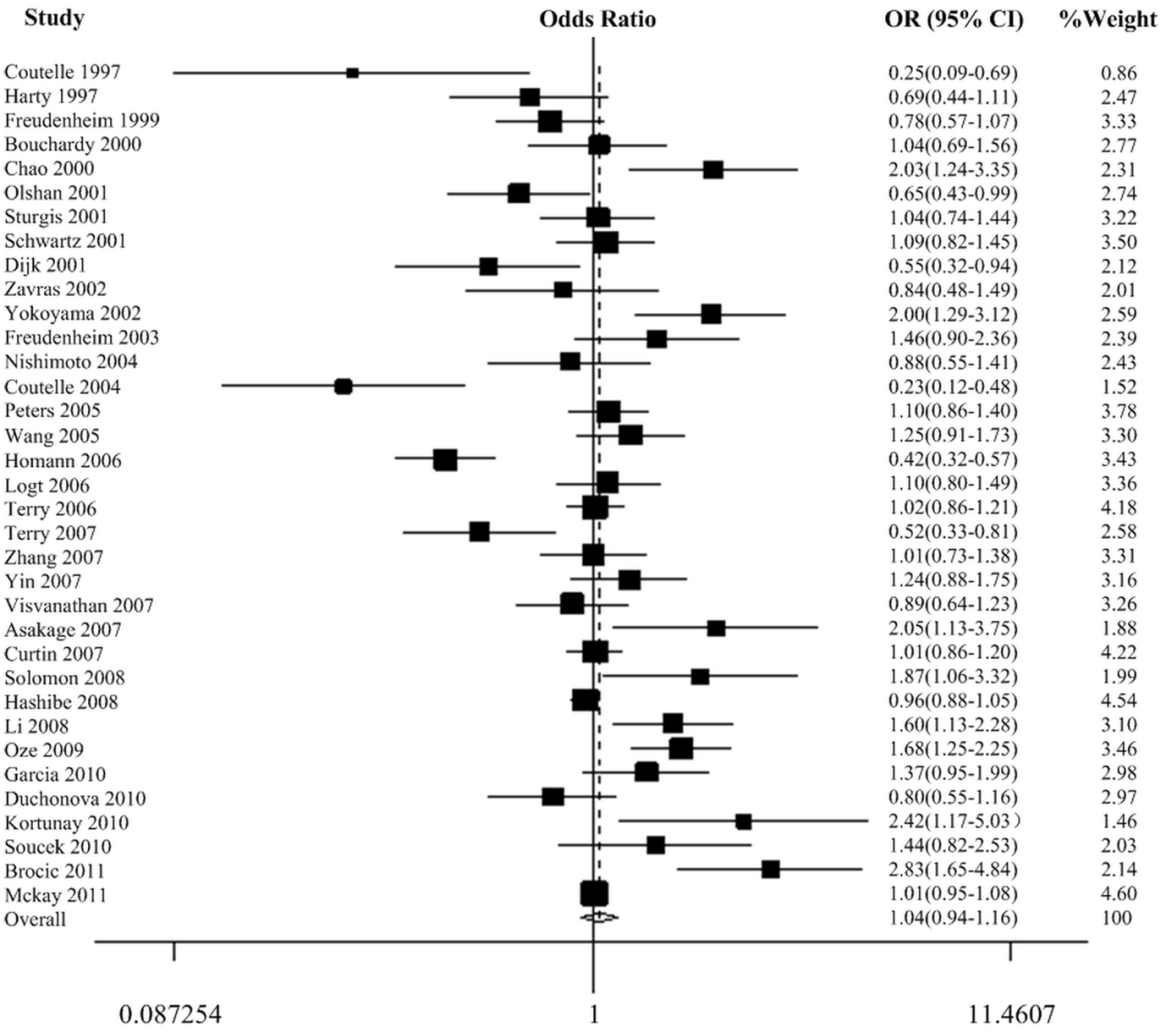
Forest plot of cancer risk associated with the *ADH1C* Ile350Val polymorphism (dominant model). The squares and horizontal lines correspond to the study-specific OR and 95% CI. The area of the squares reflects the weight (inverse of the variance). The diamond represents the summary OR and 95% CI.

**Figure 3:**
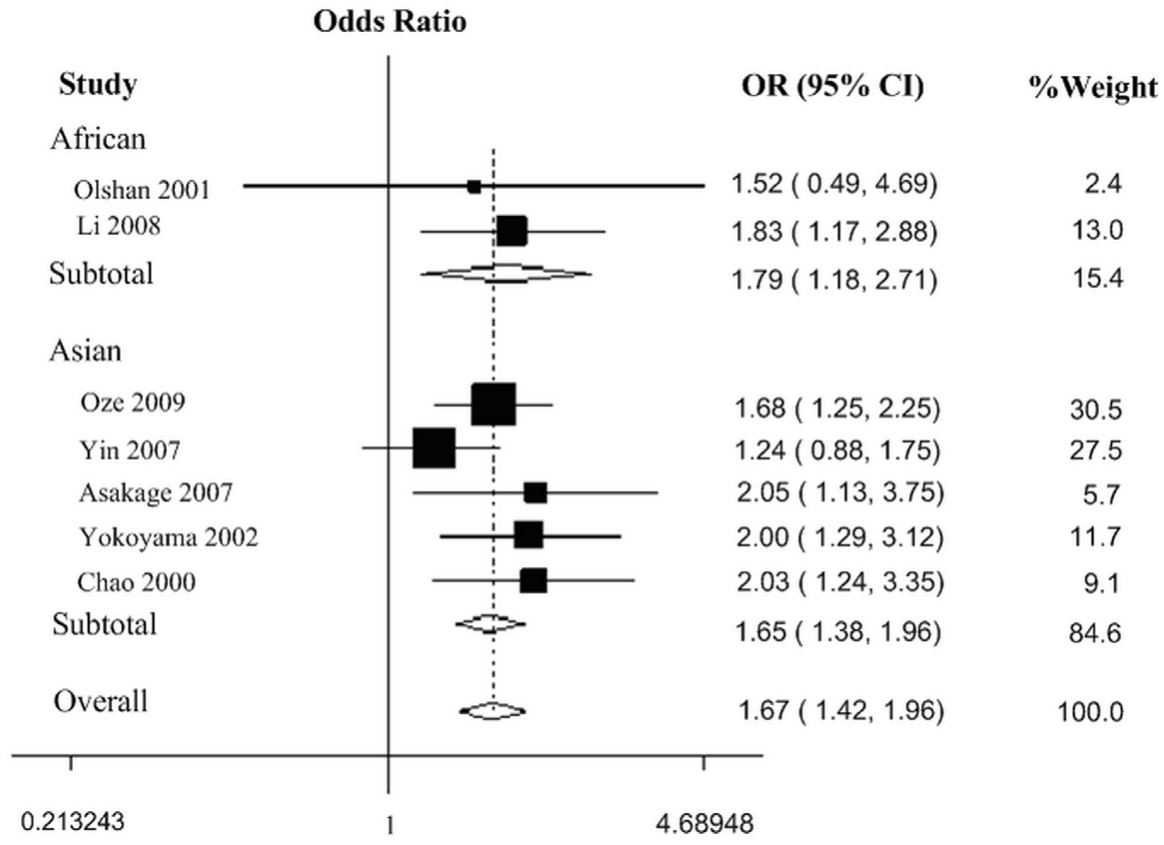
Forest plot of cancer risk associated with the *ADH1C* Ile350Val polymorphism in African and Asian populations (dominant model). The squares and horizontal lines correspond to the study-specific OR and 95% CI. The area of the squares reflects the weight (inverse of the variance). The diamond represents the summary OR and 95% CI.

There was a wide variation of the 350 Val allele frequency among the controls across different ethnicities. The 350 Val allele frequency was the lowest in Asian populations and was the highest in European populations (0.05, 95% CI  = 0.03−0.09, vs. 0.40, 95% CI  = 0.36−0.44). In African and mixed populations, the allele frequency was 0.29 (95% CI  = −1.15−1.58) and 0.35 (95% CI  = 0.27−0.39), respectively. The difference among the four population groups was statistically significant (*P*<0.001). In the overall analyses, we did not observe any significant associations between the *ADH1C* Ile350Val polymorphism and cancer risk in all the genetic models (Table 2, Fig. 2 of dominant model). Because population admixture may be a potential cause of inconsistent results [52], we excluded studies with mixed populations to further evaluate the overall effect of *ADH1C* Ile350Val polymorphism and we still didn’t find any significant associations (data not shown). However, in the stratified analysis by ethnicity, significant increased risks were found for African populations (homozygote comparison: OR  = 2.19, 95% CI  = 1.29−3.73, *P*_heterogeneity_  = 0.989; dominant model: OR  = 1.79, 95% CI  = 1.18−2.71, *P*_heterogeneity_  = 0.761; recessive model: OR  = 1.92, 95% CI  = 1.16−3.17, *P*_heterogeneity_  = 0.981) and Asian populations (heterozygote comparison: OR  = 1.58, 95% CI  = 1.32−1.90, *P*_heterogeneity_  = 0.375; homozygote comparison: OR  = 3.84, 95% CI  = 1.74−8.49, *P*_heterogeneity_  = 0.160; dominant model: OR  = 1.65, 95% CI  = 1.38−1.96, *P*_heterogeneity_  = 0.330; recessive model: OR  = 3.54, 95% CI  = 1.62−7.75, *P*_heterogeneity_  = 0.154) (Table 2, Fig. 3 of dominant model ). When we performed stratified analyses by cancer type, we found individuals with the Val/Val genotypes had a 0.58-fold lower breast cancer risk compared with the Ile/Ile genotype (OR  = 0.58, 95% CI  = 0.34−1.00, *P*_heterogeneity_  = 0.001, Table 2) (*P*  = 0.049, data not shown). We did not observe any significant associations among UADT cancer, colorectal cancer and other cancers (Table 2). However, cancer-specific analysis excluding studies with mixed populations indicated that a moderate increased risk of UADT cancer was associated with variant Val allele in dominant model (OR  = 1.17, 95% CI  = 1.01−1.36, *P*_heterogeneity_ <0.001, data not shown). Furthermore, when we conducted stratified analyses according to source of controls and sample size, no significant associations were found in any genetic models (Table 2).

**Table 2:** Stratification analyses of the *ADH1C* Ile350Val polymorphism on cancer.

Variables	n^a^	Sample size (case/control)	Ile/Val vs. Ile/Ile		Val/Val vs. Ile/Ile		Ile/Val + Val/Val vs. Ile/Ile (dominant)		Val/Val vs. Ile/Val + Ile/Ile (recessive)	
			OR (95% CI)	*P* ^b^	OR (95% CI)	*P* ^b^	OR (95% CI)	*P* ^b^	OR (95% CI)	*P* ^b^
Total	35	19154/26519	1.02 (0.92−1.14)	<0.001	1.01 (0.87−1.16)	<0.001	1.04 (0.94−1.16)	<0.001	1.03 (0.93−1.15)	<0.001
Cancer Type										
Breast	4	1782/1880	0.78 (0.54−1.12)	0.003	0.58 (0.34−1.00)	0.001	0.73 (0.50−1.07)	0.001	0.72 (0.51−1.01)	0.048
UADT	25	14903/20901	1.09 (0.95−1.24)	<0.001	1.10 (0.93−1.31)	<0.001	1.12 (0.98−1.28)	<0.001	1.09 (0.96−1.24)	0.004
Colorectal	3	1920/3131	1.05 (0.92−1.21)^c^	0.537	1.07 (0.87−1.31)^c^	0.504	1.06 (0.93−1.21)^c^	0.566	1.06 (0.88−1.27)^c^	0.445
Other	4	549/1336	0.72 (0.44−1.17)	0.008	0.76 (0.42−1.39)	0.018	0.73 (0.44−1.20)	0.003	0.93 (0.71−1.24)^c^	0.199
Race*										
African	2	204/198	1.47 (0.89−2.44)^c^	0.867	**2.19 (1.29**−**3.73)** c	0.989	**1.79 (1.18**−**2.71)** c	0.761	**1.92 (1.16**−**3.17)** c	0.981
Asian	5	1688/3657	**1.58 (1.32**−**1.90)** c	0.375	**3.84 (1.74**−**8.49)** c	0.160	**1.65 (1.38**−**1.96)** c	0.330	**3.54 (1.62**−**7.75)** c	0.154
European	21	12964/17476	0.93 (0.81−1.06)	<0.001	0.98 (0.82−1.17)	<0.001	0.94 (0.82−1.08)	<0.001	1.03 (0.91−1.17)	0.002
Mixed	10	4294/5186	1.04 (0.90−1.20)	0.052	1.05 (0.83−1.34)	0.013	1.05 (0.89−1.23)	0.006	1.02 (0.90−1.150) c	0.110
Source of controls										
PB	14	5663/7885	1.05 (0.93−1.18)	0.017	0.98 (0.83−1.17)	0.019	1.05 (0.92−1.19)	0.001	0.97 (0.88−1.08)^c^	0.116
HB	16	2866/4719	1.01 (0.79−1.29)	<0.001	1.16 (0.79−1.69)	<0.001	1.06 (0.82−1.37)	<0.001	1.24 (0.93−1.66)	0.002
Combined	5	10625/13915	0.95 (0.74−1.21)	<0.001	0.89 (0.65−1.21)	<0.001	0.93 (0.73−1.20)	<0.001	0.96 (0.82−1.12)	0.049
Sample Size^d^										
<500	28	5333/8028	1.00 (0.83−1.19)	<0.001	1.00 (0.79−1.27)	<0.001	1.02 (0.85−1.22)	<0.001	1.05 (0.89−1.23)	0.001
>500	7	13821/18491	1.04 (0.96−1.13)	0.094	1.01 (0.89−1.15)	0.069	1.06 (0.97−1.16)	0.026	1.01 (0.89−1.14)	0.049

a : Number of studies.

*P* ^b^: The value of heterogeneity test.

c : Fix-effects model was used when *P* value for heterogeneity test >0.10; otherwise, random-effects model was used.

d : Stratified according to subjects >500 in both case and control groups or not.

Combined, studies conducted on both population-based and hospital-based control group.

* The sum of sample size of each race group was less than total sample size because in Olshan’s study, the sum of sample size of each race group was less than its total sample size.

### Test for Heterogeneity

There was significant heterogeneity for heterozygote comparison (Ile/Val versus Ile/Ile: *P*_heterogeneity_ <0.001), homozygote comparison (Val/Val versus Ile/Ile: *P*_heterogeneity_ <0.001), dominant model comparison (Val/Val + Ile/Val versus Ile/Ile: *P*_heterogeneity_ <0.001) and recessive model comparison (Val/Val versus Ile/Val + Ile/Ile: *P*_heterogeneity_ <0.001). Then, we assessed the source of heterogeneity for heterozygote comparison (Ile/Val versus Ile/Ile) by ethnicity, cancer type, source of controls and sample size. As a result, ethnicity (χ^2^ = 28.01, df  = 3, *P*<0.001) and cancer type (χ^2^ = 8.39, df  = 3, *P*  = 0.039) but not source of controls (χ^2^ = 3.54, df  = 2, *P*  = 0.171) or sample size (χ^2^ = 0.52, df  = 1, *P*  = 0.470) were found to contribute to substantial heterogeneity.

### Sensitivity Analyses

Sensitivity analyses indicated that two independent studies by Hashibe *et al*. in 2008 and Homann *et al*. in 2006 were the main origin of heterogeneity [14], [50]. In addition, no other single study influenced the pooled OR qualitatively, as indicated by sensitivity analyses, suggesting that the results of this meta-analysis are stable.

Furthermore, when we performed cancer-specific and population-specific sensitivity analyses we found studies conducted by Terry *et al.* in 2006 [34], Hashibe *et al*. in 2008 [14], Homann *et al*. in 2006 [50] and Terry *et al.* in 2007 [21] were the main origin of heterogeneity in subgroup of breast cancer, UADT cancer, European population and mixed population, respectively. Moreover, no single study influenced the pooled OR in each subgroup, which indicated that results of stratified analyses were also stable.

### Publication Bias

**Figure 4:**
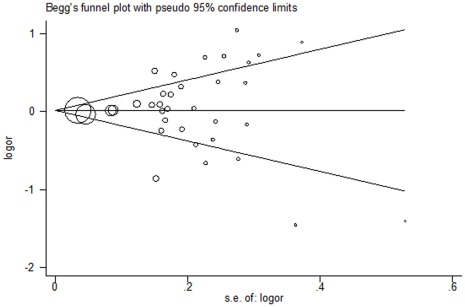
Begg’s funnel plot for publication bias test (dominant model). Each point represents a separate study for the indicated association. Log[or], natural logarithm of OR. Horizontal line, mean effect size.

Begg’s funnel plot and Egger’s test were performed to evaluate the publication bias of literatures. As shown in Fig. 4, the shape of the funnel plots seemed symmetrical in the dominant model comparison. Then, the Egger’s test was adopted to provide statistical evidence of funnel plot symmetry. The results still did not show any evidence of publication bias (t  = 0.42, *P*  = 0.674 for Val/Val + Ile/Val versus Ile/Ile).

## Discussion

Genetic variation in carcinogen metabolism pathway may exert influence on the risk of exposure-related cancer [9]. Based on the vital role of ADH1C in ethanol oxidation to AA, numerous studies have investigated the association of the functional *ADH1C* polymorphism with types of cancers. Several studies have observed significant association between ADH1C*1 allele and cancer risk [21], [24], [38], [41], [43]–[45], [48]–[50]. It has been widely known that ADH1C*1 allele encode isozymes with higher catalytic activity than the one encoded by ADH1C*2 allele and result in more production of AA, which is a major part in ethanol-related carcinogenesis [3] and alcoholism [53].

However, in view of the linkage disequilibrium between *ADH1B* and *ADH1C* and the fact that the kinetic differences among ADH1B isozymes are much more striking than those among the ADH1C isozymes [54], some studies ascribed the association between *ADH1C* polymorphism and cancer risk to the reflects of effect of *ADH1B* polymorphism, especially in East Asian [18], [32]. But a matter of particular note was the significant difference of allele frequency between *ADH1B* and *ADH1C* polymorphism, that is, the minor allele frequency (MAF) for *ADH1B* was 0–0.025 in European (ADH1B*2 allele)and 0.223–0.261 in Asian (ADH1B*1 allele) populations while the MAF for *ADH1C* was 0.473–0.483 in European (ADH1C*2 allele) and 0.023–0.081 in Asian (ADH1C*2 allele) populations (data from www. ncbi. nlm. nih. gov/projects/SNP/snp_ref. cgi), which made the general attribution of *ADH1C* effect to its linkage with *ADH1B* not reasonable. In other words, as the two more-active alleles, ADH1B*2 and ADH1C*1 were linked and the frequency of ADH1B*2 was too low in European, the explanation of ADH1C effect was not well founded, especially in European. Furthermore, there were also some studies suggesting that the influence of *ADH1C* polymorphism on cancer risk was independent of that of *ADH1B* in both European and Asian populations [14], [20], [55], [56]. Thus, the detail mechanism underlying *ADH1C* polymorphism and cancer risk remains controversial and the hypothesis that the variant of *ADH1C* exert an independent influence on cancer risk by changing ethanol oxidizing capacity [10] was better founded.

Although many studies have investigated the association between the *ADH1C* polymorphism and cancer risk, the results were inconsistent. In order to resolve this conflict, we conducted a meta-analysis of 35 case-control studies. Because data could be confounded by the differences between subgroups, we subsequently conducted stratified analysis by cancer type, ethnicity, source of controls and sample size. Moreover, as Deng *et al.* suggested in 2001 that population admixture may potentially elevate type I error rate of association studies and lead to inconsistent results [52], we also conducted overall and cancer-specific analyses excluding studies with mixed populations to confirm the effect of this polymorphism and the impact of mixed populations.

Generally speaking, we did not find any association between Ile350Val polymorphism and overall cancer risk. This result indicated that individuals with the *ADH1C* genotype leading to more exposure to acetaldehyde from alcohol were not at statistically different risk of cancers. When we further performed analyses excluding mixed populations, there were still no associations between this polymorphism and overall cancer risk. To a certain extent, analyses excluding mixed populations confirmed the negative result of initial overall analyses.

In the analysis stratified by cancer type, we still did not find any significant associations among studies of breast cancer, UADT cancer, colorectal cancer and other cancers in any genetic model. However, a similar meta-analysis reported recently had shown that ADH1C*1 allele was associated with a significantly decreased risk of pharynx cancer in dominant model [20]. Probably the discrepancy arose because they collect data of either Arg272Gln or Ile350Val polymorphism which were in perfect linkage disequilibrium but may have minor differences of genotype distribution [14], [25], [27] and relatively small number of studies (22 studies) they included. Interestingly, we found the effect of variant 350 Val allele on breast and other cancer risk was contrary to that on UADT and colorectal cancer, although all the effects were not significant. As heterogeneity among different cancers may interfere the authenticity of result in “other cancers”, the inverse result of breast cancer studies called more attention. A possible explanation is that carcinogenesis involved in different cancers is extremely diverse. Thus, specific role of ADH1C in carcinogenic mechanisms of breast cancer [27], [57] as well as interaction between special risk factors of breast cancer [58] and *ADH1C* gene may contribute to the inconsistent results.

Furthermore, when we performed cancer-specific analyses excluding studies with mixed populations, major results were nearly the same except that a moderate increased UADT cancer risk was founded in individuals carrying Val allele (i.e. dominant model). However, as the lower limit of the 95% CI was 1.01 in that comparison and the removing of some studies with relatively large sample size (although they were with mixed populations) may also decrease the reliability of result, we didn’t think this result was sufficient to support the risk effect of Val allele in UADT cancer. Therefore, studies with more samples randomly selected from one homogeneous population are needed to further determine the association between this variant and specific cancer risk.

Subsequently, we found an increased risk of cancer in variant homozygote (Val/Val) carriers among Africans and in variant allele (350 Val) carriers among Asians. Studies have indicated that 350 Val allele increases the risk for alcoholism [59], which may lead to accumulated exposure to the highly toxic and carcinogenic material, AA [4]. Thus, it is plausible that the presence of 350 Val allele puts one at a greater cancer risk through susceptibility to alcoholism. Although a few studies of Europeans suggested this variation might be significantly associated with risk of cancer [21], [41], [43], [44], [45], [49], [50], the overall difference was not significant. We presume that the difference among ethnic groups might be a reflection of different genetic backgrounds and environmental context. As a number of studies attributed the effect of *ADH1C* variant in East Asian to its linkage disequilibrium with *ADH1B*, it would be better for us to adjust the association found in Asian for *ADH1B* polymorphism. However, among the five studies conducted in Asian populations, only one [18] provided detailed data of *ADH1C* genotype adjusted for *ADH1B* genotype. Thus, the independent effect of *ADH1C* polymorphism in Asians could not be directly estimated in the present analysis, which to some extent was a flaw. In addition, other factors such as relatively small sample size (204 VS. 198 of African studies and 1688 VS. 3657 of Asian studies), selection bias and different matching criteria may also be a possible explanation to this result.

Although hospital-based studies may have inherent selection biases, we did not find any positive result in the stratified analysis by population-based and hospital-based controls, indicating that the different source of controls did not influence the association. In addition, because studies with small sample size may have insufficient statistical power or may have generated a fluctuated risk estimate, we performed stratified analyses according to subjects more than 500 in both case and control groups or not and no significant association was detected. These results suggested that there was no substantial impact of study sample size on this meta-analysis.

Because identification of the source of heterogeneity was very important in a meta-analysis, we subsequently detected source of heterogeneity by stratifying studies according to ethnicity, cancer type, source of control and sample size. Results showed the sources of heterogeneity were from ethnicity and cancer type, suggesting that certain effects of genetic variant were population and cancer specific.

Our meta-analysis had some advantages. First, substantial number of cases and controls were pooled from different studies, which significantly increased statistical power of the analysis. Second, studies included in our present meta-analysis strictly met our selection criteria. Third, we did not detect any publication bias indicating that the whole pooled result may be unbiased.

Except for the lacking of evaluation of independent effect of *ADH1C* adjusted for *ADH1B* in Asian, we also had a limitation of the present study. It has been identified that after generated from oxidization of alcohol by ADH enzymes, AA was further oxidized to acetate by aldehyde dehydrogenase (ALDH) enzymes and ALDH2 contributed most to the process [60]. Thus, besides ADH, activity of ALDH2 can also exert impact on accumulation of AA. A functional polymorphism in *ALDH2* has been identified (rs671) to be associated with cancer risk [61], [62], which lead to different activity of ALDH2 enzyme and is prevalent in Asians [60]. Although polymorphisms of *ALDH2* were not in linkage disequilibrium with *ADH*, it might influence the effect of polymorphisms of *ADH1C* through its impact on AA elimination. Therefore, *ALDH2* polymorphism was a potential confounder of the present study, especially of Asian studies. Independent and combined effect of *ADH1B*, *ADH1C* and *ALDH2* variants should be evaluated in further meta-analyses.

In conclusion, our results suggested that the *ADH1C* Ile350Val polymorphism is not a candidate for susceptibility to overall cancers. However, an increased cancer risk was observed in populations among African and Asian, but not in European and mixed race, which may be a reflection of ethnic differences. Additional larger studies assessing gene-gene and gene-environment interactions should be performed to further clarify the association of *ADH* genetic variants and cancer risk.
